# Study on the inhibitory mechanism of dehydrogenated antioxidants on coal spontaneous combustion

**DOI:** 10.1038/s41598-022-25721-1

**Published:** 2022-12-08

**Authors:** Xun Zhang, Chen Yu, Bing Lu, Fei Gao, Chuan Shan, Jiahui Zou

**Affiliations:** 1 College of Mining Engineering, Liaoning Technical University, Fuxin, 123000 Liaoning China; 2 College of Safety Science and Engineering, Liaoning Technical University, Fuxin, 123000 Liaoning China; 3 China Coal Science and Engineering Energy Technology Development Co, LTD, Beijing, 100028 China

**Keywords:** Coal, Reaction mechanisms

## Abstract

In order to comprehensively and systematically analyze the reasons why antioxidant inhibitors can scavenge free radicals in coal and inhibit coal spontaneous combustion, this paper studies the effects of VC, TBHQ, EGCG and BHT on coal spontaneous combustion by means of coal spontaneous combustion characteristics experiments and quantum chemical simulation methods. The low-temperature oxidation characteristics of coal were studied through temperature-programmed experiments. The results showed that the CO emission of coal samples with antioxidants was significantly lower than that of raw coal. At 170 °C, the maximum decrease was 37.74%. Fourier infrared test showed that compared with the coal samples without antioxidant treatment, the adsorption strength of hydroxyl structure and oxygen-containing functional groups of the treated coal samples was significantly reduced. The area percentages of hydroxyl and methylene changed significantly, decreased by 7.14% and 6.46%, respectively. Subsequently, molecular models of four antioxidants were constructed using quantum chemical theory, and their Mulliken charges, BDE values ​and frontier orbitals were calculated according to density functional theory (DFT), and the active sites and inhibition mechanisms of antioxidants were discussed. The results showed that H_9_ of VC, H_33_ of EGCG, H_1_ of TBHQ and H_40_ of BHT all had strong ability to scavenge oxygen-containing free radicals, and their order of strength was TBHQ > BHT > EGCG > VC. Antioxidant inhibitors mainly reduce the number of active free radicals by removing the peroxide groups in the initial stage of the coal oxygen reaction, and remove the hydroxyl groups to prevent the further spontaneous combustion of coal and inhibit the low temperature oxidation process of coal.

## Introduction

Coal, as an important fossil energy, may react with oxygen during various stages of mining, transportation and storage, resulting in spontaneous combustion of coal^1,2^. Spontaneous combustion of coal will not only cause great resource loss, ecological damage, but may even cause casualties^3,4^. Every year, a lot of work has been done in the prevention and control of spontaneous combustion of coal, and key technologies to control spontaneous combustion of coal have been developed, such as grouting^5^, nitrogen injection^6^ and spraying of chemical inhibitors^7^. Inhibitors are widely used because of their simple and convenient use and their remarkable inhibitory effect on coal spontaneous combustion^8^. At present, the commonly used inhibitors include inorganic salt inhibitors (chloride^9^, ammonium salt and lye, etc.) and chemical inhibitors^10^. Inorganic salt inhibitor can effectively prevent coal from contacting with oxygen due to its own physical properties, and has a certain effect on controlling spontaneous combustion of coal, but it does not start from the mechanism of spontaneous combustion of coal. According to the theory of coal spontaneous combustion free radicals^11^, coal spontaneous combustion is caused by the breaking of chemical bonds in the coal, releasing a large amount of heat, and triggering a chain cycle reaction in the coal^12^. Chemical inhibitors can inhibit the spontaneous combustion of coal by inhibiting the reactivity of coal. Ma et al.^13,14^ found that adding a mixture of ascorbic acid and acrylic acid to coal can inhibit the spontaneous combustion of coal by preventing the heat accumulation of coal. Deng et al.^15^ studied the inhibition effect of three inhibitors on spontaneous combustion of coal. The results showed that these three inhibitors can reduce the production of CO_2_ in the process of coal oxidation and have a good inhibition effect on coal oxidation. Lu et al.^16^ studied the inhibition effect of 1-butyl-3-methylimidazolium tetrafluoroborate on spontaneous combustion of coal under different oxygen concentrations. The results showed that with the increase of the concentration of the inhibitor solution, the composite combustion index of coal shows a parabolic downward trend. 2,2,6,6-Tetramethyl-1-piperidinyloxy (TEMPO) and *N*,*N*-dibenzylhydroxylamine (DBHA) were tested as coal spontaneous combustion inhibitors by Li and Wang et al.^17,18^. The results showed that TEMPO and DBHA will combine with part of the coal structure to form inert substances. Since the inert substances do not have the reactivity of coal, they will hinder the low-temperature oxidation process of coal, thereby preventing the spontaneous combustion of coal. Li et al.^10^ added antioxidants to coal and analyzed the CO, CO_2_ and other gases generated during the low-temperature oxidation of coal. The study showed that the oxidation activation energy of coal was improved after adding antioxidants, and the inhibition rate of coal oxidation could reach 73.08%. It can be seen that the chemical antioxidant type inhibitor can play an excellent inhibitory performance on coal spontaneous combustion.

In order to comprehensively and systematically analyze the mechanism of coal spontaneous combustion inhibition by antioxidant type inhibitors, TBHQ, EGCG, BHT, VC are selected as typical antioxidant type inhibitors in this paper. The inhibition effects of four antioxidants on coal low-temperature oxidation are compared and analyzed by means of experiment and simulation. The inhibition effects of four different antioxidants were analyzed and compared through low-temperature oxidation experiment with CO release amount as index^19^. The effect of antioxidants on active groups in coal was analyzed by Fourier infrared spectroscopy^20^. The charge point group, bond dissociation energy (BDE) and frontier molecular orbital (LOMO–HOMO) were analyzed using density functional theory^21^ (DFT) of quantum chemical method, and the inhibition mechanism of antioxidant was obtained, thus revealing the mechanism of antioxidant inhibitor scavenging free radicals in coal and inhibiting coal spontaneous combustion.

## Experimental

### Preparation of coal samples

The coal sample used in the experiment is lignite, which had a low degree of metamorphism and was prone to spontaneous combustion. The newly exposed coal was collected together and vacuum sealed to prevent it from oxidizing before the experiment. The coal samples used in the experiment were crushed and screened under nitrogen atmosphere. The particle size of the coal sample used in the experiment is 100 mesh. After crushing and screening, the coal sample is dried for 24 h in a vacuum environment at 40 °C. The proximate analysis is shown in Table 1. Due to the better spontaneous combustion tendency of erosive coal, the erosive coal which is easier to spontaneous combustion than raw coal is used as the research object to add antioxidant to carry out the oxidation inhibition experiment of coal. The structures of the antioxidants are listed in Table 2. The coal and water were soaked in a ratio of 1:5 for 30 days, and the eroded coal sample was obtained by vacuum drying after filtration. Then 3.0 g of the different antioxidants EGCG and VC were dissolved in 20.0 g of deionized water, respectively. Since BHT and TBHQ are water-insoluble organic substances, 3 g of BHT and TBHQ were respectively dissolved in the same amount of 50% ethanol. 50 g of the prepared eroded coal sample was evenly mixed with the antioxidant solution, sealed and stored at room temperature for 24 h, and then vacuum-dried to constant weight. The same amount of deionized water was added to the control coal sample without antioxidants, and then the same treatment was performed to ensure the validity and objectivity of the experiment. Therefore, raw coal samples without antioxidants and comparison coal samples with antioxidants were prepared.

**Table 1:** Proximate analysis.

Proximate analysis (%)					Elemental analysis (%)				
Coal	Moisture	Volatile matter	Ash	Fixed carbon	C	H	O	N	S
Lignite	8.36	30.84	20.26	40.54	69.22	3.87	24.67	1.06	1.18

**Table 2:** Four antioxidant structures.

Name	VC (Vitamin C)	EGCG ((–)-Epigallocatechin gallate)	BHT (Butylated hydroxytoluene)	TBHQ (Tert-butyl hydroquinone)
Molecular structure				

### CO concentration experiment

**Figure 1:**
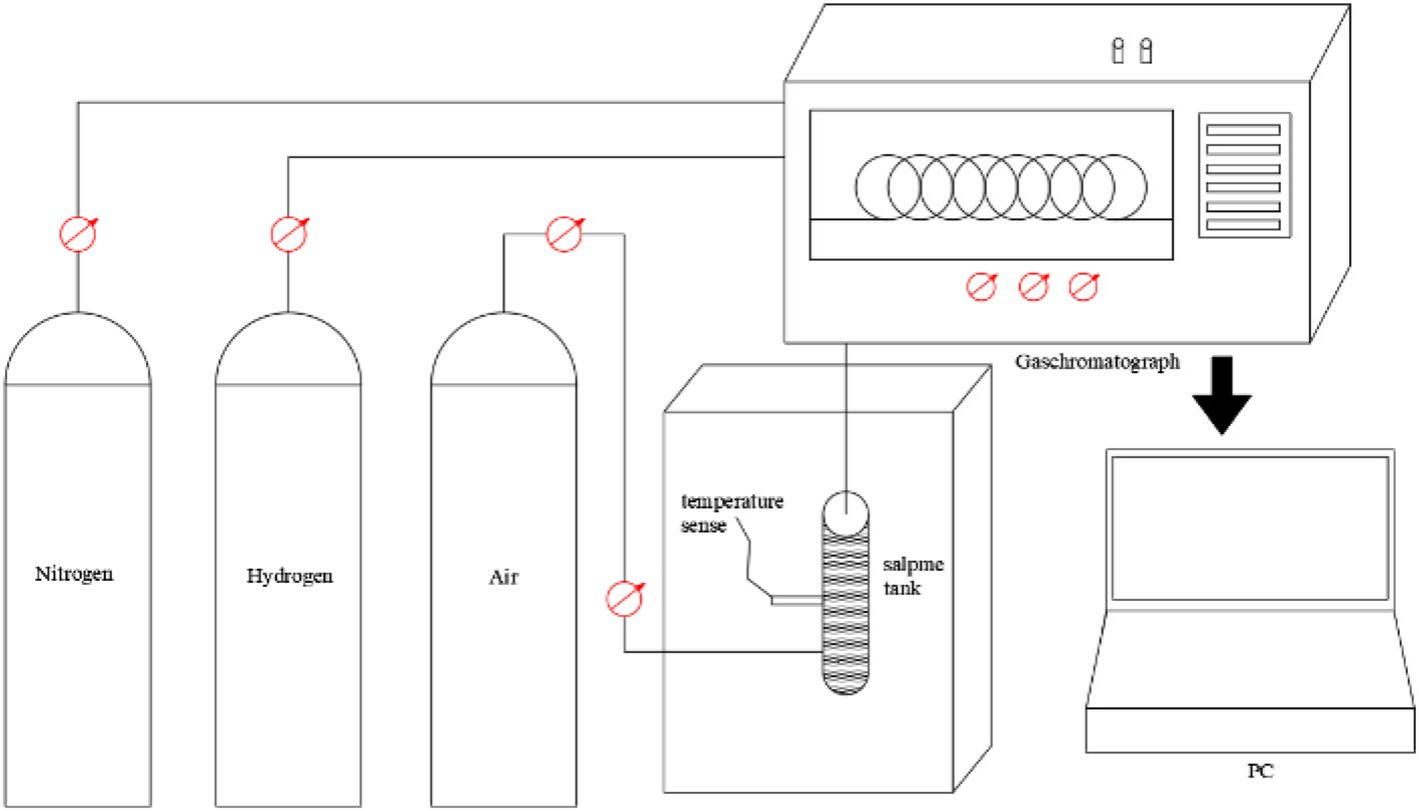
Coal sample temperature-programmed experimental setup (ZRD briquette oxidation temperature tester).

The amount of CO produced during low-temperature oxidation can be used as an important indicator to determine the degree of spontaneous combustion of coal, and it can be tested by a combination of a temperature-programmed experimental device and a chromatographic analysis device. The device is shown in Fig. 1. The prepared coal sample is put into the programmed heating device, and the programmed heating is started. The air flow rate into the equipment is set to 100 ml/min, the heating rate of the equipment is set to 0.8 °C/min, and the test temperature range is set to 30–180 °C. The heated coal sample was kept at 30 °C for 15 min and the gas was tested once. The composition and concentration of the gas are measured by gas chromatograph every 10 °C.

### Infrared spectrum experiment

The German TENSOR27 Fourier infrared spectrometer was used in the experiment. Raw coal and anti-oxidant-added coal samples were heated to 100 °C and then cooled in a vacuum environment. Then put the prepared coal sample and potassium bromide in a mortar and grind it in a ratio of 1:180. After grinding, the mixed powder was put into the HY-15 tablet press for tableting treatment. Pressure was applied for 30 s to produce samples with good uniformity and light transmittance. The experimental wave range was 400–4000 cm^−1^ with 64 scans. Blank backgrounds were collected before each experiment, and the resulting data were baseline-corrected for spectra and smoothed.

### Theoretical calculation of antioxidant scavenging free radicals

So far, density functional theory (DFT) has been widely used in the study of molecular structure and properties. In this paper, four antioxidants VC, BHT, TBHQ and EGCG were selected, and the molecular structure model of four antioxidants and active oxygen-containing free radicals in coal was constructed by GaussView6.0. To further elucidate the reaction behavior of antioxidant molecules, the molecular structure was optimized using the B3LYP/6-311G(d,p) basis set, and charge population analysis was performed. The BDE of the O–H group was subsequently calculated and the reactivity of antioxidant molecules and radicals was analyzed based on the frontier orbitals of the optimized molecular structure model. The orbits were calculated at the level of B3LYP/6-311G(d, p), and the Gaussian software was used for all calculations.

## Results and discussion

### CPT analysis

CPT is the balance point between external heat absorption and internal heat release of the coal sample. It can be used to reflect the degree of coal oxidation difficulty. In this paper, the CPT of coal samples with different antioxidants was tested and analyzed. The results are shown in Table 3.

**Table 3:** The CPT of raw coal and the antioxidant coal sample.

Coal samples	Raw coal	Coal + VC	Coal + EGCG	Coal + BHT	Coal + TBHQ
CPT (°C)	130.4	136.6	138.9	146.7	147.2

It can be seen from the data in the table that the CPT of coal after adding different antioxidants is higher than that of raw coal. The CPT of the coal sample with VC is 136.6 °C, which is 6.2 °C higher than that of the raw coal. After adding EGCG, the CPT of coal samples increased by 8.5 °C compared with that of raw coal. The CPT of the coal sample with BHT increased by 16.3 °C compared with the raw coal. The CPT of the coal sample with TBHQ increased by 16.8 °C compared with the raw coal. After the addition of antioxidants in coal, CPT increases to varying degrees, indicating that antioxidants have an inhibitory effect on the oxidation of coal. The coal samples containing BHT and TBHQ showed better inhibition effect.

### CO emission analysis

**Figure 2:**
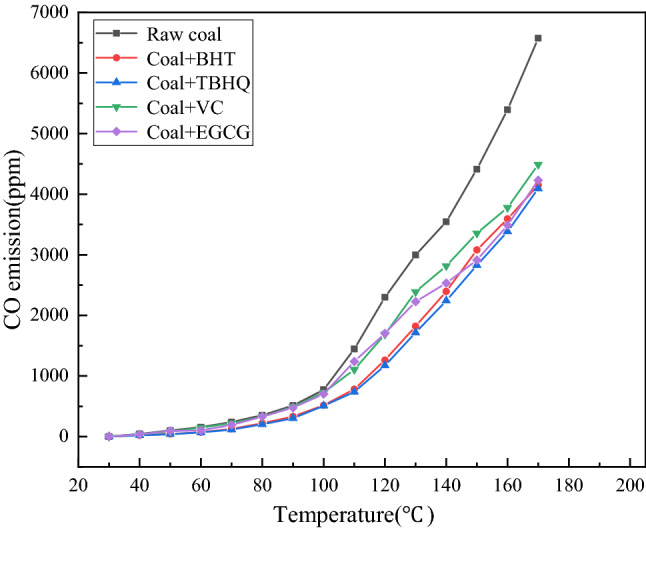
Concentration of CO released from coal samples with temperature.

In the process of coal spontaneous combustion, CO will appear as one of the products when the coal temperature exceeded 40 °C. The coal spontaneous combustion free radical theory believes that CO is the product of the reaction between free radicals and oxygen in coal, so the concentration of CO can intuitively reflect the reaction intensity of free radicals^22^. In this paper, the CO emissions of four antioxidants (EGCG, BHT, VC, TBHQ) and raw coal were compared through experiments, and the CO emissions curves of coal samples before and after inhibition were obtained. As shown in Fig. 2.

From Fig. 2, The measured CO emission of the coal sample with antioxidant is significantly lower than that of the raw coal. By comparing and analyzing the CO concentration curves of the oxidation process of coal samples treated with four antioxidants, it is found that the CO emissions of VC and EGCG are less than that of raw coal, while the CO emissions of BHT and TBHQ are less than that of VC and EGCG. The CO emissions of the coal samples treated with antioxidants at 170 °C were reduced by 31.71%, 35.64%, 36.87% and 37.74%, respectively, compared with the raw coal. This is because they reduced the concentration of free radicals in coal during low temperature oxidation of coal. Decrease the rate of free radical chain cycle reactions. Therefore, the CO emission of coal samples treated with antioxidants is reduced to different degrees, which also reflects the relationship between the strength of antioxidant inhibition, namely TBHQ > BHT > EGCG > VC.

### Infrared spectroscopic analysis

**Figure 3:**
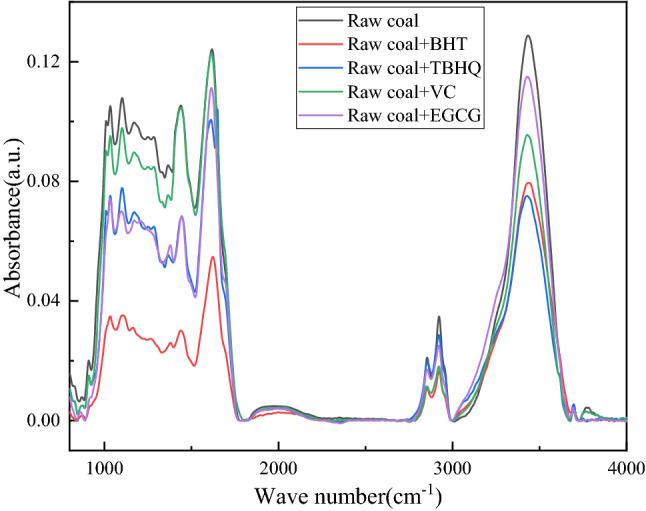
Infrared spectrum.

In the process of low temperature oxidation of coal, some weak chemical bonds in coal are destroyed by heat, forming active free radicals, resulting in the increase of the total amount of active functional groups in coal. Then the oxidation reaction of free radicals and the hydrogen extraction reaction occur^23^. FTIR can directly and specifically analyze the active functional groups^24^ in coal, so as to judge the inhibitory effect of antioxidants on coal, and it is a common technology to study coal spontaneous combustion. In order to specifically study the changes of functional groups in coal before and after antioxidant inhibition, the FTIR spectra of coal samples heated at 100 °C before and after four antioxidants (EGCG, BHT, VC, TBHQ) were tested. As shown in Fig. 3. The changes in the hydroxyl structure, aliphatic and oxygen-containing functional groups of the reactive groups correspond to the regions 3650–3000, 3000–2800 and 1800–1000 cm^−1^ in the figure, respectively. Compared with the coal samples without antioxidant treatment, the adsorption strength of the treated coal samples in the hydroxyl structure and oxygen-containing functional groups was significantly reduced, and the coal samples treated with TBHQ and BHT changed significantly. This showed that the number of functional groups in coal samples treated with antioxidants is reduced, which reflects the inhibitory effect of antioxidants on coal low-temperature oxidation.

**Figure 4:**
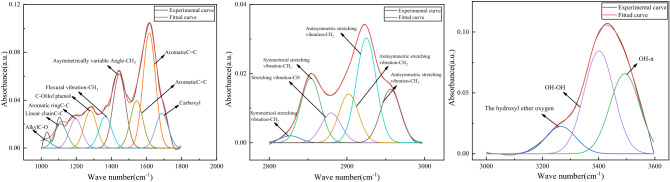
Multimodal fit curves of coals treated without antioxidants in the 3650–3000, 3000–2800 and 1800–1000 cm^−1^ regions.

**Figure 5:**
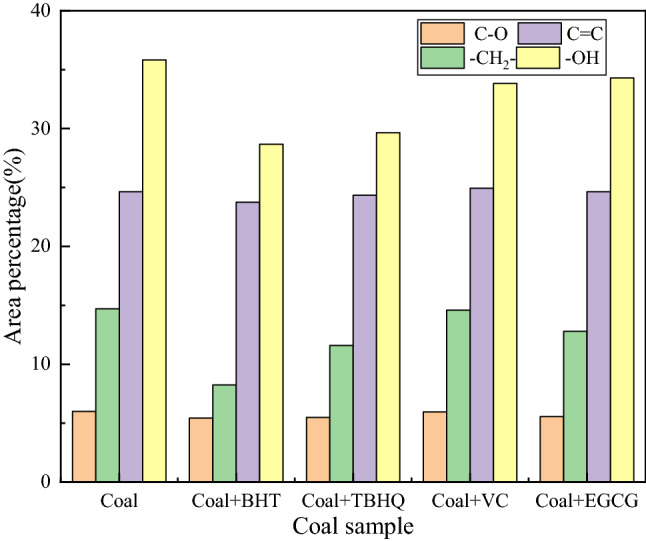
Relative content of functional groups in coal treated with different antioxidants.

To further analyze the inhibitory effect, Peak Fit was used to perform curve fitting processing on the corresponding wave function region. The changes of reactive groups in coal can be quantitatively described by fitting peak areas. The confidence coefficients of the fitted curves were all greater than 0.97. Figure 4 is the fitting curve diagram of raw coal in the 3650–3000, 3000–2800 and 1800–1000 cm^−1^ regions. Figure 5 shows the area percentage changes of the four functional groups in coal. As can be seen from Fig. 5, the area percentages of hydroxyl and methylene groups in coal samples treated with antioxidants were significantly reduced. Among them, the area percentage of BHT and TBHQ treatment changed significantly, and the hydroxyl and methylene groups were reduced by 7.14%, 6.17%, 6.46%, and 3.12%, respectively, indicating that antioxidants can effectively remove some functional groups in coal. The area percentage changes of C=C and C–O are small, because the aromatic C=C in the functional group is very stable on the benzene ring in the low-temperature oxidation stage of coal, and it is not easy to be oxidized. The C–O–C content did not change significantly, which was related to the etherification reaction between antioxidants and hydroxyl groups, and the ether bond C–O–C in coal was relatively stable. The spectral data obtained from the above experiments can directly show that antioxidants can play a very good role in inhibiting spontaneous combustion of coal.

### Antioxidant inhibition mechanism analysis of coal spontaneous combustion

#### Charge layout analysis

Many studies have showed that the hydroxyl group in antioxidants is the active site involved in the reaction and is the main donor of H atoms. The process of antioxidant scavenging free radicals is mainly based on the transfer mechanism of hydrogen atoms. Therefore, the reactivity of hydroxyl groups in different antioxidant molecules can be compared by calculating DFT^25,26^.

**Figure 6:**
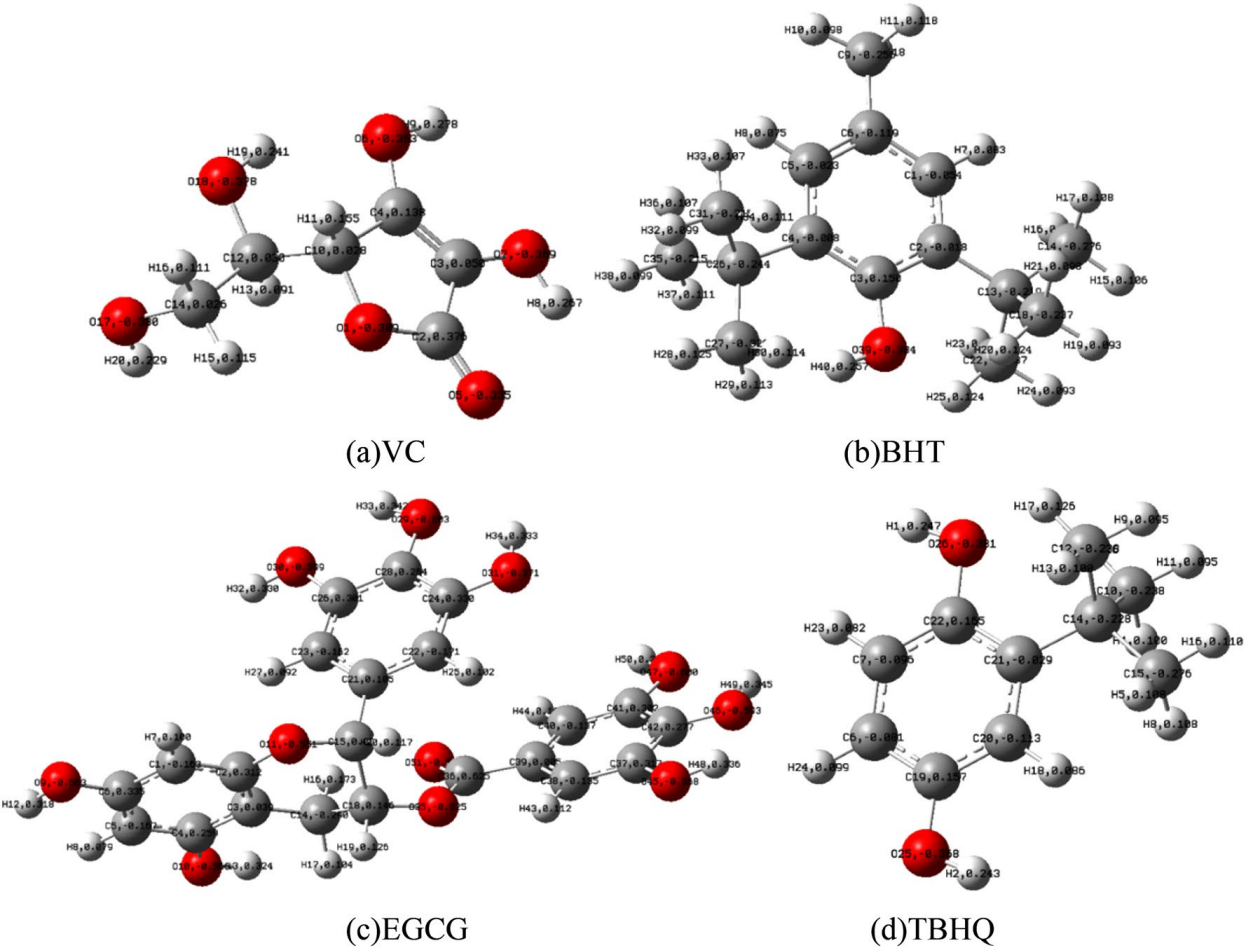
The Mulliken charges of the four antioxidants.

The Mulliken charge distributions of the four antioxidants are shown in Fig. 6. Table 4 describes the charge distribution after optimization of the molecular model, the H–O bond length in the molecule and the charge of the H atom. The H_8_–O_7_ = 0.96879 Å, H_9_–O_6_ = 0.96607 Å in the VC molecule are obviously larger than other H–O bond lengths, the atom H_9_ has the most charge of 0.278e, and the cumulative charge on the VC lactone ring is higher than the side chain. Similarly, the H_33_, H_34_, H_48_ and H_49_ atoms on EGCG contain more positive charges, and the H–O bond length is longer and has higher activity. Such analysis results are in line with the general law, and the hydroxyl ortho position has high free radical scavenging activity. Because the hydroxyl ortho position can stabilize the free radical through intramolecular hydrogen bonding. There are two para hydroxyl groups in the TBHQ molecule, and the number of charges 0.247e carried by H_1_ is greater than 0.243e carried by H_2_. There is only one hydroxyl structure in BHT, the H–O bond length is 0.96040 Å, and the positive charge of H_40_ is 0.315e. Due to the electron-pushing effect of the ortho-alkyl side chain, it is easy to lose hydrogen to generate a stable free radical B–O.

**Table 4:** Hydroxyl H–O bond lengths and accumulated charges on H atoms in four antioxidant molecules.

Antioxidants	Bond length				Charge population			
	Assignments	Value (Å)	Assignments	Value (Å)	Atoms	Charge	Atoms	Charge
VC	O_7_–H_8_	0.96879	O_18_–H_19_	0.96369	H_20_	0.229	H_8_	0.267
	O_6_–H_9_	0.96607	O_17_–H_20_	0.96198	H_19_	0.241	H_9_	0.278
EGCG	O_9_–H_12_	0.96588	O_31_–H_34_	0.96912	H_12_	0.318	H_34_	0.333
	O_10_–H_13_	0.96573	O_47_–H_50_	0.96515	H_13_	0.324	H_50_	0.331
	O_30_–H_32_	0.96525	O_46_–H_49_	0.96885	H_32_	0.330	H_49_	0.345
	O_29_–H_33_	0.96835	O_45_–H_48_	0.96911	H_33_	0.342	H_48_	0.336
TBHQ	O_26_–H_1_	0.96196	O_25_–H_2_	0.96210	H_1_	0.247	H_2_	0.243
BHT	O_39_–H_40_	0.95765			H_40_	0.257		

The H atoms on the H–O bonds in the four antioxidant molecules all have a large number of positive charges, while the oxygen atoms in the active radicals in coal are usually negatively charged and tend to bond with the positively charged hydrogen atoms. Therefore, the antioxidant properties of the four antioxidants are mainly due to the presence of highly reactive hydroxyl groups. Therefore, the longer the length of the H–O bond, the more positive charges of the H atom in the H–O bond, and the easier it is to have a hydrogen extraction reaction with the free radical.

#### Bond dissociation energy analysis

The scavenging process of free radicals under meteorological conditions is mainly through the transfer of H atoms, which is largely controlled by H-OBDE. Therefore, in order to further clarify the reaction sites of antioxidants, the value of BDE was calculated to reflect the free radical scavenging activity of antioxidants. The calculation formula is: BDE = H_298K_(RO·) + H_298K_(H·) − H_298K_(ROH). Among them, H_298K_(RO·), H_298K_(H·) and H_298K_(ROH) represent the enthalpy of radical RO·, the enthalpy of dehydrogenation to H· at 298 K and the enthalpy of ROH molecule, respectively, kcal/mol. The lower the value of BDE, the weaker the strength of the chemical bond and the easier it is to break. Geometry optimization and frequency calculations were performed for VC, BHT and TBHQ at the B3LYP/6-311G(d,p) level. However, due to the large number of atoms in EGCG, the computational cost is high, and the calculation is performed at the B3LYP/6-31G(d,p) level^27^. All quantum chemical calculations were performed using Gaussian software. According to the above analysis of charge and bond length, the four hydroxyl groups on VC were calculated respectively. The activity of EGCG is mainly based on the hydroxyl groups related to H_33_, H_34_, H_48_, and H_49_, so the BDE calculation of the four O–H is carried out. TBHQ contains two active hydroxyl groups and BHT contains one active hydroxyl group, and the BDE calculations are carried out respectively. By compared the H-OBDE values of the four antioxidant molecules, the sequence of H–O bond cleavage can be determined. The H-OBDE results of the calculated model molecules are shown in Table 5. For VC, the order of BDEs is H_19_–O_18_ > H_20_–O_17_ > H_8_–O_7_ > H_9_–O_6_, which also indicates that H_9_-O_6_ in the VC molecule is easy to break first. Also for EGCG, H_33_-O_29_ tended to break first, as reflected by the values of BDEs. The reaction sequence of the two hydroxyl groups of TBHQ is H_1_ > H_2_. The hydroxyl group of BHT, the value of BDE is 75.0501 kcal/mol. The order of H atom activity of antioxidants was not completely consistent with the results of bond length and charge analysis. However, the analysis results all showed that H_9_ of VC, H_33_ of EGCG, H_1_ of TBHQ, and H_40_ of BHT have strong hydrogen-donating ability and have a positive effect on scavenging free radicals^17^.

**Table 5:** H-OBDE of model molecules.

Enthalpy	H (RO, Hartree)	H (H, Hartree)	H (ROH, Hartree)	BDE (Hartree)	BDE (kcal/mol)
**VC**					
H_8_–O_7_	− 684.2051	− 0.4998	− 684.8302	0.1253	78.6270
H_9_–O_6_	− 684.2140	− 0.4998	− 684.8302	0.1164	73.0421
H_19_–O_18_	− 684.1700	− 0.4998	− 684.8302	0.1604	100.6526
H_20_–O_17_	− 684.1740	− 0.4998	− 684.8302	0.1564	98.1425
**EGCG**					
H_34_–O_31_	− 1675.7419	− 0.4979	− 1676.3666	0.1268	79.5683
H_33_–O_29_	− 1675.7507	− 0.4979	− 1676.3666	0.1180	74.0461
H_48_–O_45_	− 1675.7340	− 0.4979	− 1676.3666	0.1347	84.5255
H_49_–O_46_	− 1675.6279	− 0.4979	− 1676.3666	0.2408	151.1044
**TBHQ**					
H_1_–O_26_	− 539.2822	− 0.4998	− 539.9020	0.1200	75.3012
H_2_–O_25_	− 539.2778	− 0.4998	− 539.9020	0.1244	78.0622
**BHT**					
H_40_–O_39_	− 660.5418	− 0.4998	− 661.1612	0.1196	75.0501

#### Orbital analysis

**Figure 7:**
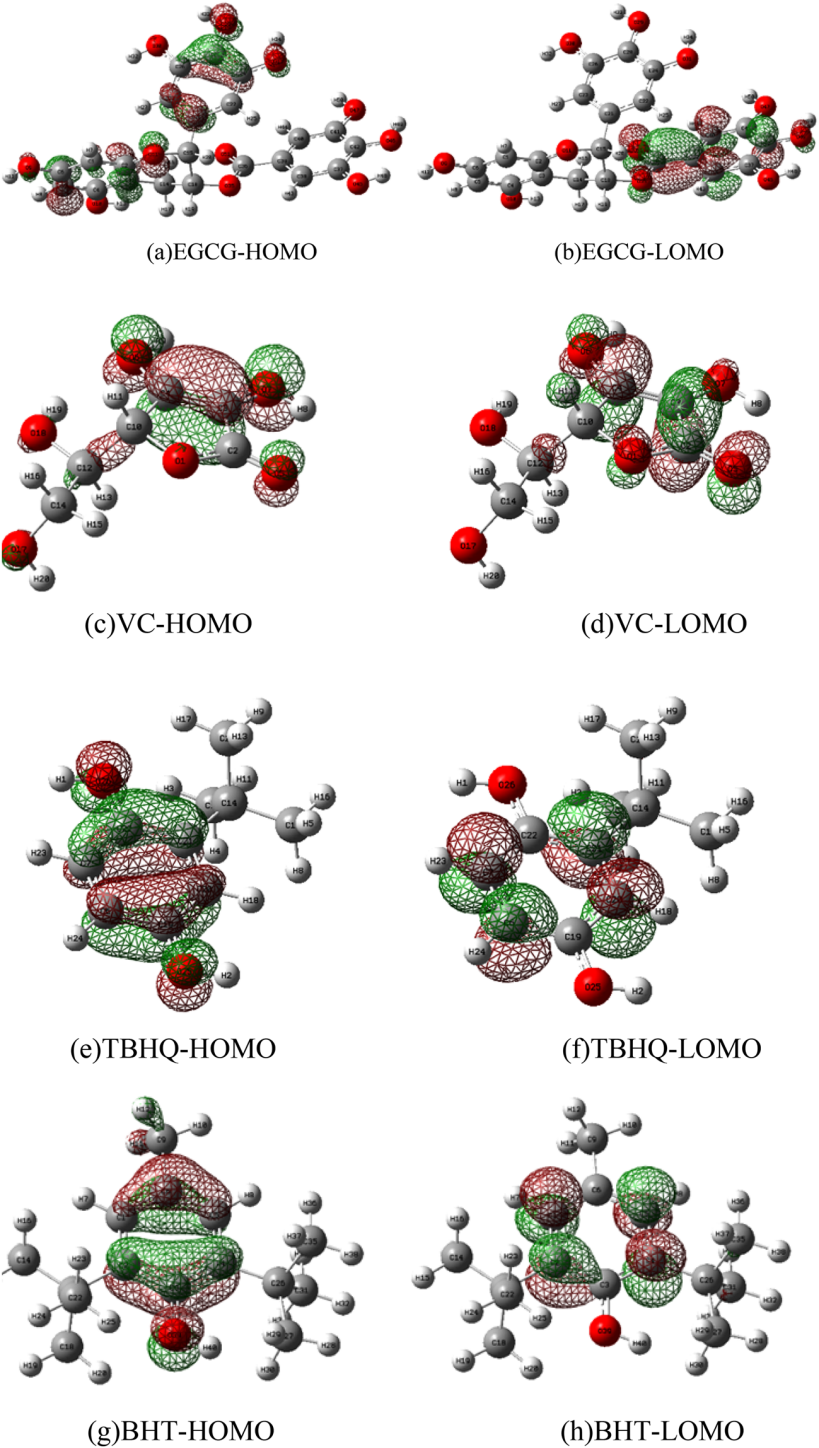
Frontier molecular orbitals of antioxidants.

Using molecular orbital theory to further analyze the degree of difficulty and ease of antioxidants in inhibiting the reaction process of oxygen-containing functional groups. The HOMO and LUMO energies of antioxidants and oxygen-containing functional groups were calculated at the B3LYP/6-311G(d,p) level, as shown in Fig. 7. HOMO and LUMO orbitals are the dominant orbitals in the chemical reaction process, HOMO energy represents the ability to donate electrons, and LUMO energy represents the ability to gain electrons. The difference between the two values represents the energy gap (LUMO–HOMO), which can reflect the chemical reactivity of antioxidant molecules. Antioxidants stabilize free radicals by transferring one H atom through the reaction of hydrogen extraction, i.e. the HOMO as electron donor loses one electron. The HOMO of VC molecule is about H_9_–O_6_ and C_3_=C_4_, the HOMO of EGCG molecule is about H_33_–O_29_, and the HOMO of TBHQ and BHT is mainly concentrated on their hydroxyl groups, which are considered as reaction sites.

**Figure 8:**
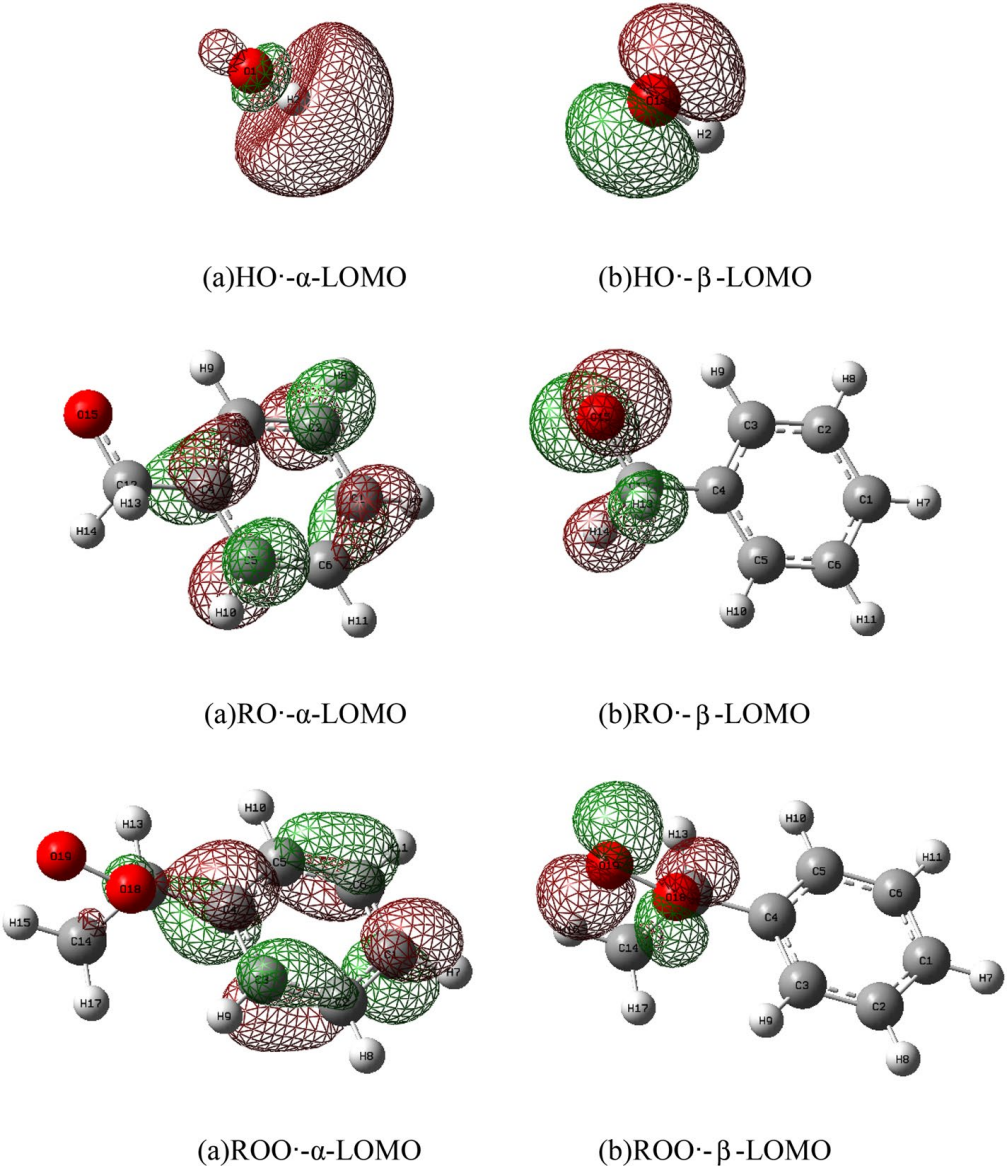
α-LOMO and β-LOMO of free radicals.

A large number of free radicals will be produced during the low-temperature oxidation of coal. In this paper, three key oxygen-containing radicals, hydroxyl HO·, alkoxy radical RO· and peroxy radical ROO· were selected for computational analysis. Since the benzene ring has little effect on the activity of free radicals, a single benzene ring was used for modeling^28^. For the radical model with weakly interacting unpaired electrons, the confinement open shell of DFT was used for the calculation. The HOMO orbital of the antioxidant loses an electron through the hydrogen extraction reaction, while the LOMO orbital of the radical gains an electron and becomes stable. The calculated oxygen-containing radicals were at the level of B3LYP/6-311G + g(d,p). The α-LOMO and β-LOMO of radicals are shown in Fig. 8, and the corresponding energy gaps are listed in Table 7. The Alpha MOs and Beta MOs orbitals contained in free radicals were analyzed. The β-LUMOs of all oxygen-containing radicals are symmetrically distributed around the oxygen-containing functional groups, and the β-LUMOs of HO·, RO· and ROO· are relatively symmetrical to the HOMO of antioxidants. However, most α-LUMOs with oxygen-containing functional groups are distributed around the benzene ring, which is difficult to participate in the reaction. From the frontier molecular orbital perspective, the symmetry is satisfied. When a chemical reaction occurs, electrons flow from the HOMO of the antioxidant to the β-LUMO of the radical. It can be seen from Tables 6 and 7 that the HOMO and LUMO orbital energy differences of the four antioxidants interacting with oxygen-containing functional groups are similar (energy gap < 6.0 eV). The smaller the energy gap, the easier it is for electrons to be excited, which is more conducive to the occurrence of chemical reactions. The hydroxyl group has the smallest energy gap difference between antioxidants and oxygen-containing radicals, which are 2.0454 eV, 1.0269 eV, 0.7468 eV, and 0.6964 eV, respectively. Followed by RO· 2.8807 eV, 1.8622 eV, 1.5821 eV, 1.5317 eV. Then ROO 3.8746 eV, 2.8561 eV, 2.5760 eV, 2.5256 eV. The orbital energies of the four antioxidants and oxygen-containing functional groups are similar (energy gap < 6.0 eV). This indicates that antioxidants can scavenge active oxygen-containing free radicals in coal very well, and the values of antioxidants and hydroxyl groups are the smallest, indicating that antioxidants will preferentially react with hydroxyl groups. According to the above data, it can be concluded that the activity sequence of scavenging free radicals is TBHQ > BHT > EGCG > VC, and the ability of BHT and TBHQ to scavenge free radicals is stronger than that of EGCG and VC. This is relatively consistent with the experimental conclusion that all three oxygen-containing radicals can be scavenged by free radicals, especially HO· and antioxidants have the smallest energy gap and are easily scavenged by antioxidants^29^.

**Table 6:** Antioxidant frontier orbital levels (HOMO, LUMO) and energy gap (eV).

Orbitals	EGCG	VC	BHT	TBHQ
HOMO (eV)	− 6.0931	− 7.1133	− 5.8130	− 5.7626
LOMO (eV)	− 1.5911	− 1.4966	− 0.2654	− 0.5272
ΔE (eV)	4.5019	5.6167	5.5475	5.2354

**Table 7:** Frontier orbital energy levels (HOMO, LUMO) and energy gap (eV) of oxygen-containing radicals.

Orbitals	RO·		HO·		ROO·	
	Alpha	Beta	Alpha	Beta	Alpha	Beta
HOMO (eV)	− 7.0293	− 7.1010	− 9.7012	− 8.9622	− 7.2717	− 7.1535
LOMO (eV)	− 0.7527	− 4.2309	0.6434	− 5.0662	− 0.9269	− 3.2370
ΔE (eV)	6.2765	2.8701	10.3446	3.8960	6.3447	3.9165

**Figure 9:**
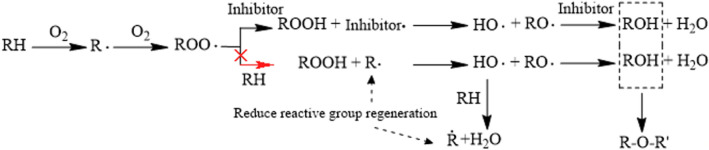
Reaction mechanism of antioxidants to eliminate oxygen-containing radicals during coal oxidation.

The four antioxidants inhibit the oxidation process of coal, and the mechanism is shown in Fig. 9. The peroxidation structure (ROO·) produced by the chemical adsorption of coal and oxygen is an important structure of coal-oxygen reaction. ROO· will react with RH in coal to form R· easy to combine with oxygen and ROOH with unstable structure. ROO· will react with RH in coal to form R· easy to combine with oxygen and ROOH with unstable structure. Due to the instability of the ROOH structure, it is easy to generate HO· and RO·, and HO· is very active and easily reacts with active groups such as ROH, RH and RCOOH in coal to generate RO·, R· and RCOO·, and enter the chain cycle. Accelerates the low temperature oxidation process of coal^30^. However, antioxidants can provide a large number of H atoms to combine with HO· and RO· to form alcohol and water, and alcohol can further stabilize the structure by forming ether structure, prevent further chain cycle reactions from occurring. In conclusion, the addition of antioxidants prevented the further oxidation of the active free radical ROO· and the further progress of the hydroxyl chain reaction^25,31^. Therefore, antioxidants can effectively eliminate the oxygen containing free radicals in the low-temperature oxidation process of coal, hinder the chain cycle reaction of free radicals in the low-temperature oxidation process of coal, and thus inhibit the further oxidation of coal.

## Conclusion

The low temperature oxidation characteristics of coal were analyzed macroscopically and microscopically by temperature programmed and infrared spectroscopy experiments, and the inhibitory effect of antioxidants on coal low temperature oxidation was studied. The reaction mechanism of four antioxidants to eliminate active oxygen-containing free radicals in the process of coal low-temperature oxidation was discussed by means of quantum chemical calculation. The main conclusions are as follows.

The four antioxidants all showed different degrees of inhibition during the low temperature oxidation of coal. CO emission reflects the inhibition effect of antioxidants. The CO emission of the coal samples treated with antioxidants was significantly lower than that of the raw coal. Among them, EGCG, TBHQ and BHT showed better inhibitory effect, and the inhibitory effect of VC was weak.

After added antioxidant, the content of hydroxyl group and oxygen-containing functional group in coal decreased obviously. Among them, the area percentages changed significantly after BHT and TBHQ treatment, and the hydroxyl and methylene groups were reduced by 7.14%, 6.17% and 6.46%, 3.12%, respectively. The scavenging of active groups in coal by antioxidants reflects the inhibitory effect on the low-temperature oxidation process of coal.

The reactivity of antioxidants and active radicals in coal was compared in terms of Mulliken charge, bond dissociation energy and frontier orbital through quantum chemical theoretical calculations. The analysis showed that the hydroxyl groups contained in the four antioxidants were the main active sites for scavenging active free radicals. Among them, the orbital energy gap of TBHQ and BHT is smaller, and the reactivity of scavenging active radicals in coal is the highest. The O–H bonds of the hydroxyl groups contained in the four antioxidants are easily broken, thus providing a large number of H atoms. These hydrogen atoms effectively scavenge the highly active hydroxyl groups and other oxygen-containing radicals in the coal, prevent the further oxidation of the active groups in the coal and the occurrence of the free radical chain cycle reaction, thereby inhibiting the low-temperature oxidation process of the coal.

## Data Availability

The datasets used and/or analysed during the current study available from the corresponding author on reasonable request.
